# Molecular mechanisms of programmed cell death in methamphetamine-induced neuronal damage

**DOI:** 10.3389/fphar.2022.980340

**Published:** 2022-08-17

**Authors:** Dongming Guo, Xinlei Huang, Tianqing Xiong, Xingyi Wang, Jingwen Zhang, Yingge Wang, Jingyan Liang

**Affiliations:** ^1^ Institute of Translational Medicine, Medical, Yangzhou University, Yangzhou, China; ^2^ Department of Neurology, Affiliated Hospital of Yangzhou University, Yangzhou, China

**Keywords:** apoptosis, autophagy, necroptosis, pyroptosis, ferroptosis, methamphetamine

## Abstract

Methamphetamine, commonly referred to as METH, is a highly addictive psychostimulant and one of the most commonly misused drugs on the planet. Using METH continuously can increase your risk for drug addiction, along with other health complications like attention deficit disorder, memory loss, and cognitive decline. Neurotoxicity caused by METH is thought to play a significant role in the onset of these neurological complications. The molecular mechanisms responsible for METH-caused neuronal damage are discussed in this review. According to our analysis, METH is closely associated with programmed cell death (PCD) in the process that causes neuronal impairment, such as apoptosis, autophagy, necroptosis, pyroptosis, and ferroptosis. In reviewing this article, some insights are gained into how METH addiction is accompanied by cell death and may help to identify potential therapeutic targets for the neurological impairment caused by METH abuse.

## 1 Introduction

### 1.1 METH

“METH” is also called “ice” or “crystal” and it is one of the most common amphetamine-type stimulants abused (Jones et al., 2020). According to statistics, nearly 27 million people used METH in 2019 and projections indicate an 11% increase by 2030 in global (UNODC, 2020). In addition to being highly addictive, this stimulant drug poses grave concerns to public health around the world, and it will take considerable research and effort to develop effective intervention strategies to combat METH’s neurotoxic side effects. In epidemiological and clinical studies, METH is a new type of drug, different from traditional drugs such as Opium, Heroin, cocaine, and marijuana, which are more addictive and more harmful to users and society (Zweben et al., 2004; Sekine et al., 2006). METH is shown to be a strong central nervous system stimulant that has a detrimental impact on human behavior, cognition, and physical health (Schep et al., 2010; Kevil et al., 2019). Taking METH in high doses can result in euphoria, delusions, hypersexuality, hyperthermia, cardiac arrhythmias, heart attacks, and kidney failure (Glasner-Edwards and Mooney, 2014; Baradhi et al., 2019). The effects of long-term use of the drug can be permanent insomnia, brain damage, heart failure, and schizophrenia (Dean et al., 2013; Thanos et al., 2016; El Hayek et al., 2020). In addition to being violent, METH addicts are prone to violent attacks, sexual assaults, robbery, and other crimes (Sekine et al., 2006). These factors make them a hidden danger to social security (Dean et al., 2013; Lappin et al., 2018; Mizoguchi and Yamada, 2019). Abstinence leads to depression, anxiety, fatigue, and intense craving during withdrawal (Zweben et al., 2004; Sekine et al., 2006). This neuropsychiatric complication is most commonly associated with METH-induced neurotoxicity. Continual use of METH in animals and humans has been shown to cause neurodegeneration of dopaminergic and serotonergic cells in the striatum, cortex, and hippocampi (Zhu et al., 2016; Golsorkhdan et al., 2020). There are several mechanisms by which METH causes neurotoxicity, including direct damage to terminals, oxidative stress, mitochondrial dysfunction, neuronal excitability, endoplasmic reticulum stress, and neuroinflammation, resulting in dopaminergic neuron death (Yang et al., 2018; Kim et al., 2020). Numerous studies have shown that neuronal death caused by METH exposure is closely related to the PCD signaling pathway, such as apoptosis, necroptosis, pyroptosis, and ferroptosis, as well as neuronal death (Huang et al., 2019; Wen et al., 2019). The PCD pathway is critical to METH-induced neurotoxicity, so agents that can influence the pathway may be essential components in future therapeutic strategies.

### 1.2 PCD

PCD is a cell death process mediated by molecular programs regulated by specific genes in the cell and plays a crucial role in the development process and homeostasis maintenance of multicellular organisms (Cookson and Brennan, 2001). In 1842, Carl Vogt observed toad cells, he first discovered that the body can promote growth and development by inducing cell death autonomously (Heasman et al., 1994). He defined the structural characteristics of different types of cell death by electron microscopy observation (Kerr, 1969). Coulson et al. (1986) revealed the genetic molecular mechanism of PCD by studying nematodes. So far, at least five major PCDs have been discovered and studied in recent years, including apoptosis, autophagy, necroptosis, pyroptosis, and ferroptosis. Different forms of PCD can be distinguished by their unique morphological, biochemical, or molecular features (Table 1 Illustrates morphological and biochemical hallmarks that highlight fundamental differences in pathways). Since most of the key genes involved are evolutionarily conserved, some effector molecules are often used as specific evaluation indicators of PCD. These molecules can also detect and analyze different PCD processes together with morphological and biochemical detection methods.

**TABLE 1:** Pathways of cell death and associated morphological and biochemical hallmarks.

Cell death pathway	Biochemical and morphological characteristics and core targets	
Apoptosis	Cleavage and cascade activation of various caspases; eversion of phosphatidylserine on membranes; DNA fragmentation	Hacker (2000); Saraste and Pulkki (2000)
	The shrinkage of cells, membrane blebbing, chromosome condensation, and nuclear fragmentation, eventual phagocytosis by phagosomes	
	Caspase-3, Caspase-9, PARP,Caspase-8, Caspase-12,Caspase-2, Bcl-2, Bax, Cytochrome C, p53etc.,	
Autophagy	LC3 lipidation; increased lysosomal activity	Parzych and Klionsky, (2014)
	A large accumulation of double-membrane autophagosomes in the cytoplasm; autophagosomes fuse with lysosomes to degrade their contents	
	LC3, ULK1, Beclin-1, Atg-5, Atg-12, p62, Bnip3etc.	
Necroptosis	RIPK1, RIPK3, and MLKL are phosphorylated and activated; RIPK1 and RIPK3 form a complex; MLKL multimers in cell membranes and mediates cellular pores; ATP levels decrease	Holler et al. (2000)
	Cell and organelle expansion; chromosome condensation; membrane rupture and release of cytoplasmic contents	
	RIPK1, RIPK3, MLKLetc.,	
Pyroptosis	Activation and assembly of inflammasomes; activation of pro-inflammatory caspases by cleavage; cleavage of gasdermin family members, multimerization of N-terminal fragments, and mediation of cellular channels	Rogers et al. (2019)
	Cell membrane rupture; inflammatory cytokines and cellular contents are released	
	Caspase-1, NLRP3, AIM2, ASC/TMS1, IL-1β, IL-18, Gasdermin Detc.	
Ferroptosis	Decreased levels of GSH and NADPH; inhibition of GPX4; iron accumulation and lipid peroxidation; increased levels of COX-2; depletion of polyunsaturated fatty acid phospholipids; increased ROS; production of byproduct 4-HNE.	Dixon et al. (2012)
	Mitochondrial density is less than normal mitochondria, mitochondrial outer membranes rupture, and mitochondrial cristae are reduced or disappear	
	GPX4, NRF-2, COX-2, LSH, LOXs, NOXs, SLC7A11, GSH Homeostasis regulators, carbohydrate and lipid regulatorsetc.,	

Normal development and tissue homogeneity depend on carefully regulated PCD signaling events in multicellular organisms. During embryogenesis, PCD-eliminating cells are necessary for the adequate shaping of certain tissues, such as the sculpting of vertebrate limb fingers (Haanen and Vermes, 1996; Fuchs and Steller, 2011)*.* The central nervous system (CNS), which consists of the brain and spinal cord, is shaped by PCD. The establishment of neural structures is tightly regulated by signaling events at the temporal and spatial levels. The neuronal damage caused by methamphetamine and other addictive drugs is multifactorial (Dong et al., 2009; Soto and Pritzkow, 2018). Substantial evidence supports that cell death plays a crucial role in the pathogenesis of drug addiction-induced neuronal injury. However, it remains unrevealed whether defects in cell death signaling and neuronal cell death are responsible for these diseases, and how different PCD pathways or processes interact with each other to these pathology`s death of neuronal cells.

## 2 PCD signaling pathways and their roles in METH-induced neurotoxicity

### 2.1 Apoptosis and METH neurotoxicity

Apoptosis, the first discovered form of programmed cell death, is activated by specific death signaling pathways under physiological or pathological conditions. It can remove unwanted or abnormal cells from tissues (Fuchs and Steller, 2011). Morphologically, apoptotic cells exhibit cytoplasmic shrinkage, plasma membrane budding, phosphatidylserine eversion, chromatin condensation, and DNA fragmentation (Hacker, 2000; Saraste and Pulkki, 2000). During the whole process of apoptosis, the cell membrane remains intact with no contents released. It would not cause the body’s inflammatory response (Hengartner, 2001)*.*

Apoptosis is a delicate and complex cascade regulation process. Apoptosis occurs through both extrinsic pathways (death receptor pathway) and intrinsic pathways (mitochondrial pathway). Both two pathways are participated and interconnected in the regulation of apoptosis (Igney and Krammer, 2002). Extrinsic pathways are initiated by cell surface death receptors and related ligands, such as FasL/Fas, TNF/TNFR1, Apo3L/DR3, Apo2L/DR4, and Apo2L/DR5 are among the ligands and death receptors involved (Suliman et al., 2001; Rubio-Moscardo et al., 2005). Fas-related protein (FADD/TRADD) recruits caspases-8/10, forming death-inducing signaling complexes (DISC), where caspase-8 is oligomerized and activated by autocatalysis, and the activated caspase, in turn induce downstream cascades (Boatright and Salvesen, 2003). Activated caspase-8/10 cleaves other caspases (caspase-3/6/7) precursors into activated forms of caspases. These activated, executive-phase caspases lead to the degradation of various cellular structural components such as the cytoskeleton and nucleus. When caspase-8 is inhibited or blocked, RIPK1 and RIPK3 interaction lead to autophosphorylation, transphosphorylation, and assembly of the “necroptosis complex” (composed of RIPK1, RIPK3, and MLKL), which further initiates an alternative form of PCD, or programmed necrosis (Cho et al., 2009; He et al., 2009).

Endogenous apoptotic pathways are triggered by intracellular stress or damage signals (e.g., endoplasmic reticulum stress, oxidative stress, DNA damage), converge at the mitochondrial level, and initiate downstream signaling cascades (Green and Kroemer, 2004; Kroemer et al., 2007). Subsequently, The activated pro-apoptotic members of the Bcl-2 family (such as Bax, Bak, etc.,) neutralize the anti-apoptotic proteins Bcl-2, Bcl-xL, and Mcl-1, resulting in disruption of mitochondrial outer membrane permeability (MOMP), and release of Cytochrome c (Danial and Korsmeyer, 2004). Cytochrome c binds and activates Apaf-1 and pro-caspase-9 to constitute “apoptotic bodies” (Hill et al., 2004), which then activate caspases (caspase-3/6/7) in the executive phase, leading to the degradation of various cellular substrates and apoptosis (Kuribayashi et al., 2006).

#### 2.1.1 Mitochondria damage-induced apoptosis and METH-neurotoxicity

**FIGURE 1:**
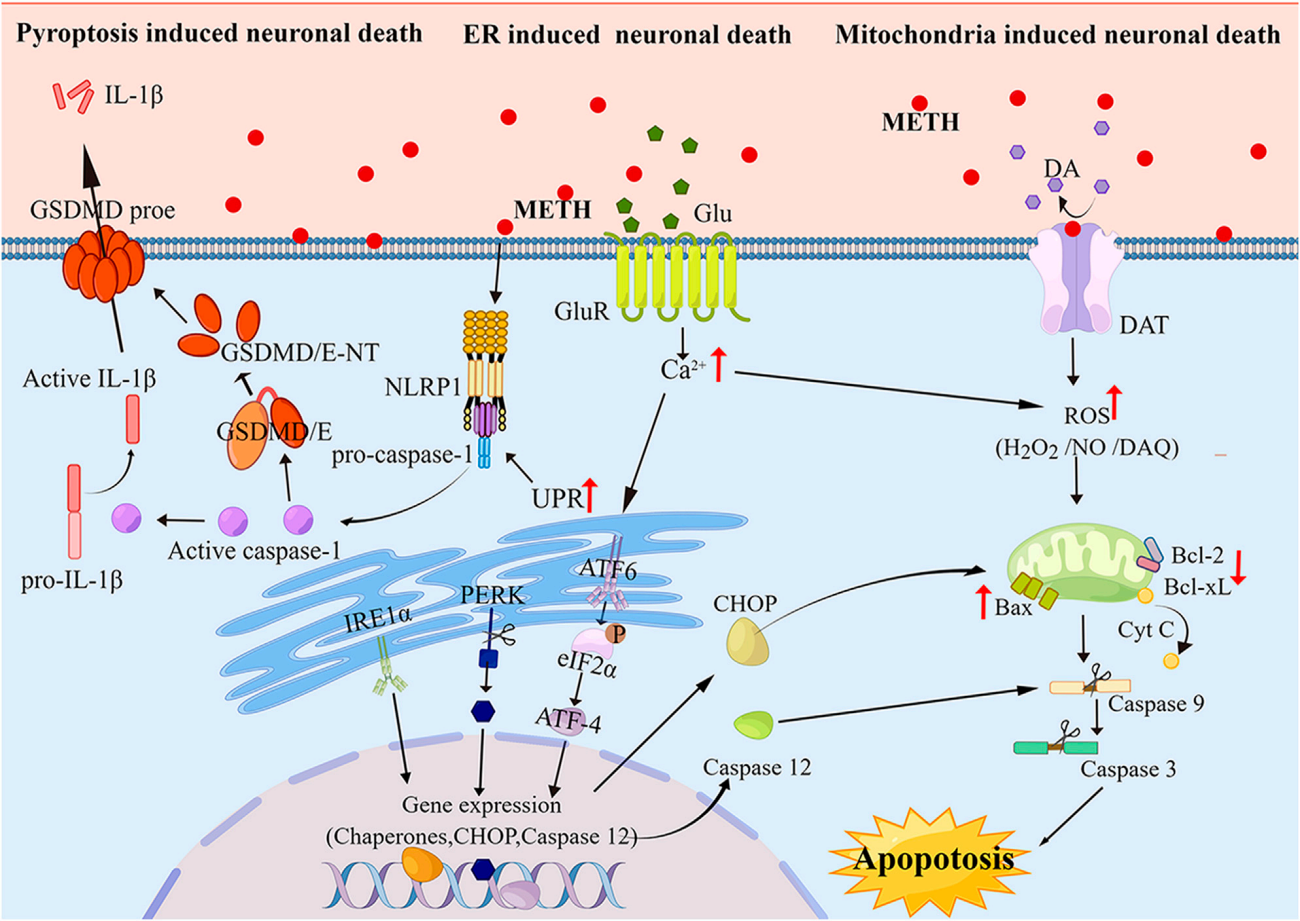
The Molecular pathways of apoptosis and pyroptosis induced by METH Neurotoxicity As METH induces apoptosis and pyroptosis via neurotoxicity pathways, it produces ROS and Ca2^+^ that act as secondary messengers for mitochondria- and ER-mediated apoptosis and pyroptosis. Furthermore, ER stress can activate the pyroptotic pathway, leading to the cleavage of GSDME, subsequently cleaving the cytoplasmic membrane and releasing cellular contents.

Neurotoxicity caused by METH is largely related to mitochondrial dysfunction (Cadet et al., 2005; Cadet et al., 2007). Autoxidation of cytosolic and extracellular dopamine (DA) results in the production of DA quinone and reactive oxygenspecies (ROS) (Cadet and Brannock, 1998). The neurological and psychiatric manifestations of other disorders, such as Parkinson’s and schizophrenia, including neuropathology, may also involve similar mechanisms (Berman and Hastings, 1999; Jayanthi et al., 2005). Inflammation of the mitochondria is one of the side effects of DA oxidation, causing swelling of the mitochondria and the opening of the permeability transition pore (Jayanthi et al., 2005; Khan et al., 2005; Jana et al., 2011). Exposed individuals to METH exhibit increased levels of pro-apoptotic proteins such as Bax and Bad and decreased levels of anti-apoptotic proteins such as Bcl-2 and Bcl-xL (Jayanthi et al., 2001; Jayanthi et al., 2004; Beauvais et al., 2011). By METH, apoptotic proteins are increased by the release of mitochondrial proteins in the intramembrane space (IMS), such as apoptosis-inducing factor (AIF) and cytochrome c (Deng et al., 2002; Jayanthi et al., 2004; Galluzzi et al., 2009). Inducing neuronal cell death by caspase-9 and caspase-3 is achieved by the release of both AIF and the second mitochondria-derived caspase activator/IAP-binding protein (SMAC/DIABLO) (Cadet et al., 2005; Shin et al., 2018). It is essential for the caspase-dependent mitochondrial apoptotic pathway to release cytochrome induced by cytochrome c, which forms an apoptosome consisting of Apaf-1, d ATP, and procaspase-9, these caspases are sequentially activated to cause apoptosis (Dang et al., 2016; Shin et al., 2018). Cytochrome C has been shown to increase the mitochondrial release and subsequent caspase activation after METH exposure (Jayanthi et al., 2004; Beauvais et al., 2011; Dang et al., 2016). METH`s degenerative effects can be mitigated with melatonin, an antioxidant (Wisessmith et al., 2009). Additionally, according to another study, METH toxicity is associated with the activation of caspase-3 and poly ADP-ribose polymerase (PARP) in the brain (Dang et al., 2016). As a result, these results suggest that METH regulates mitochondrial pathways in the brain, to influence neuronal apoptosis (Figure 1).

#### 2.1.2 ER stress-induced apoptosis and METH neurotoxicity

According to growing evidence, METH-induced cell death can also be mediated by endoplasmic reticulum (ER) stress (Krasnova and Cadet, 2009; Yu et al., 2015; Yang et al., 2018). In addition to regulating protein folding and calcium signaling, the ER is a very important organelle for maintaining cellular homeostasis (Ferri and Kroemer, 2001)*.* Stress and apoptosis can be caused by dysregulation of calcium homeostasis in the ER (Paschen, 2001)*.* It is thought that some proteases can be activated by calcium-mediated cell death, including those that cleave actin and fodrin (Mashima et al., 1999; Utz and Anderson, 2000). METH induces cellular neurotoxicity and ER stress, which are necessary for maintaining cellular homeostasis (Jayanthi et al., 2004). Recently observed calcium dysregulation and ER stress have been implicated in METH-induced cellular apoptosis (Stephans and Yamamoto, 1994; Mark et al., 2004). There is evidence that METH increases the release of glutamate in the brain’s striatum, the main excitatory neurotransmitter. The accumulated Glu binds to AMPAR and NMDAR, where it initiates downstream signaling pathways leading to a rise in Ca^2+^ levels (Halpin et al., 2014; Kim et al., 2020). The ER is responsible for controlling Ca^2+^ levels by sequestering it. Ca^2+^ is one of the most important intracellular signal transducers. Induced by METH, excess intracellular Ca^2+^ disturbs intracellular Ca^2+^ concentration, activates protein kinases and phosphatase, phosphatase, and nitric oxide synthase (NOS), and eventually results in ER stress (Bahar et al., 2016; Moratalla et al., 2017). When stressed, protein folding capacity in the ER decreases, which leads to the accumulation of unfolded and misfolded proteins in the ER, and the unfolded protein response (UPR) takes place to remove these proteins and maintain ER homeostasis (Go et al., 2017). The processes affecting UPR are mediated by protein kinase-RNA-like endoplasmic reticulum kinase (PERK), activating ATF6 as well as inositol-requiring transmembrane kinase/endonuclease 1 (IRE1) (Khanna et al., 2021; Shacham et al., 2021; Siwecka et al., 2021; van Anken et al., 2021). C/EBP homologous protein (CHOP) is known to trigger UPR-dependent apoptosis (Sano and Reed, 2013; Bahar et al., 2016). CHOP is a transcription factor that upregulates Bax and Bak expression and downregulates Bcl-2 and Bcl-XL expression to promote apoptosis (Hu et al., 2018). The elevated Ca^2+^ activates calpain, a calcium-activated neutral cysteine endopeptidase that is found in the cytosol (Nakagawa and Yuan, 2000). In the cytosol, calpain is activated, and it translocates to the membrane where it cleaves procaspase-12 (Martinez et al., 2010). However, the active caspase-12 triggers positive feedback stimulation by activating caspase-9 and caspase-3 to potentiate apoptosis (Figure 1).

METH-mediated ER stress is found to be dependent on the activation of DA receptors in the rat brain (Beauvais et al., 2011; Cadet and Bisagno, 2015). Hence, similar agents could help counteract the toxicity of the drug in humans. It has also been observed that toxic doses of methamphetamine significantly increase the expression of PERK and caspase-12 and that these effects can be diminished by inhibiting cyclin-dependent kinase 5(CDK5), an enzyme t phosphorylates Tau (Hashiguchi et al., 2002). Moreover, Liu et al. (Liu et al., 2020) found that METH signaled through three ER stress pathways in hippocampal neuronal cells (HT-22) in a time- and dose-dependent manner. The disruption of ER functions induced by METH is accompanied by altered ER calcium homeostasis (Chen et al., 2019). A marker of ER stress, secreted ER calcium monitoring proteins (SER CaMPs) are induced by the loss of ER calcium, which also increased significantly by METH (Henderson et al., 2014; Chen et al., 2019). hat.

### 2.2 Autophagy and METH neurotoxicity

In autophagy, eukaryotic cells degrade their damaged organelles and proteins with lysosomes by regulating autophagy-related genes (Atg) (Agarraberes and Dice, 2001). Autophagy prevents cellular damage, promotes cell survival under nutrient deprivation, and responds to cytotoxic stimuli (Bandyopadhyay et al., 2008). There are two types of autophagy: basal autophagy in physiological conditions and induced autophagy under stress (He and Klionsky, 2009). Cells use the latter as a means of self-protection, resulting in better growth and development (Nikoletopoulou et al., 2015). The lipids that make up the cell membrane help cells deal with metabolic stress and oxidative damage, and they also play an important role in maintaining cellular health and in synthesizing, reducing, and recycling cellular components (Kaushik et al., 2006; Nakai et al., 2007). Excessive autophagy, however, can result in metabolic stress, cellular degradation, and even cell death (Kiffin et al., 2004).

According to the different packaging materials and transport methods, in general, autophagy can be divided into three different types: macroautophagy, microautophagy, and chaperone-mediated autophagy (CMA) (Mizushima et al., 2010). A macro autophagy process (hereafter called autophagy) is an intracellular degradation reaction by which proteins, macromolecules, and cytoplasmic organelles are degraded, and invading pathogens and cellular debris are degraded in the lysosomes to build building blocks for cell renewal and homeostasis (Mizushima and Komatsu, 2011; Parzych and Klionsky, 2014). The process of autophagy begins in the cytoplasm with the formation of a pre-autophagosomal structure and evolves into phagophores, followed by autophagosomes, an organelle-containing double-membrane vacuole denatured and containing damaged macromolecules (Hansen and Johansen, 2011). After which, the outer membrane of autophagosome fuses with that of lysosome to form autolysosome, and the inner membrane and substances encapsulated in autophagosomes enter the lysosomal cavity, where they are degraded by activated lysosomal hydrolases (Klionsky and Emr, 2000). The autophagic process is highly conserved, containing multiple consecutive events regulated by proteins associated with autophagy (Meijer and Codogno, 2004; Mizushima, 2007). An initial step in the process involves the formation of a phagophore that may be triggered by various cellular stresses, including starvation and hyperthermia (Chu et al., 2008). Regulation of this process is mediated by the phosphoinositide 3-kinase (PI3K)-Beclin-1-Atg14-Vps15 complex (Klionsky, 2007). The next step is the formation of the autophagosome, where the phagophore expands to absorb the cytosolic component (Sanchez-Martin and Komatsu, 2020). Atg12-Atg5 and LC3 complexes are involved in this step of autophagy (Sanchez-Martin and Komatsu, 2020). As a result of the fusion of autophagosomes with lysosomes, cytosolic components are degraded (Gatica et al., 2018).

In recent studies, it has been shown that many drugs with abuse liability cause autophagy, including cocaine (Cao et al., 2016), heroin (Kovacs et al., 2015), morphine (Zhao et al., 2010), tetrahydrocannabinol (Hung et al., 2009) and nicotine (Hung et al., 2009). METH (Larsen et al., 2002) have also been shown to induce autophagy.

**FIGURE 2:**
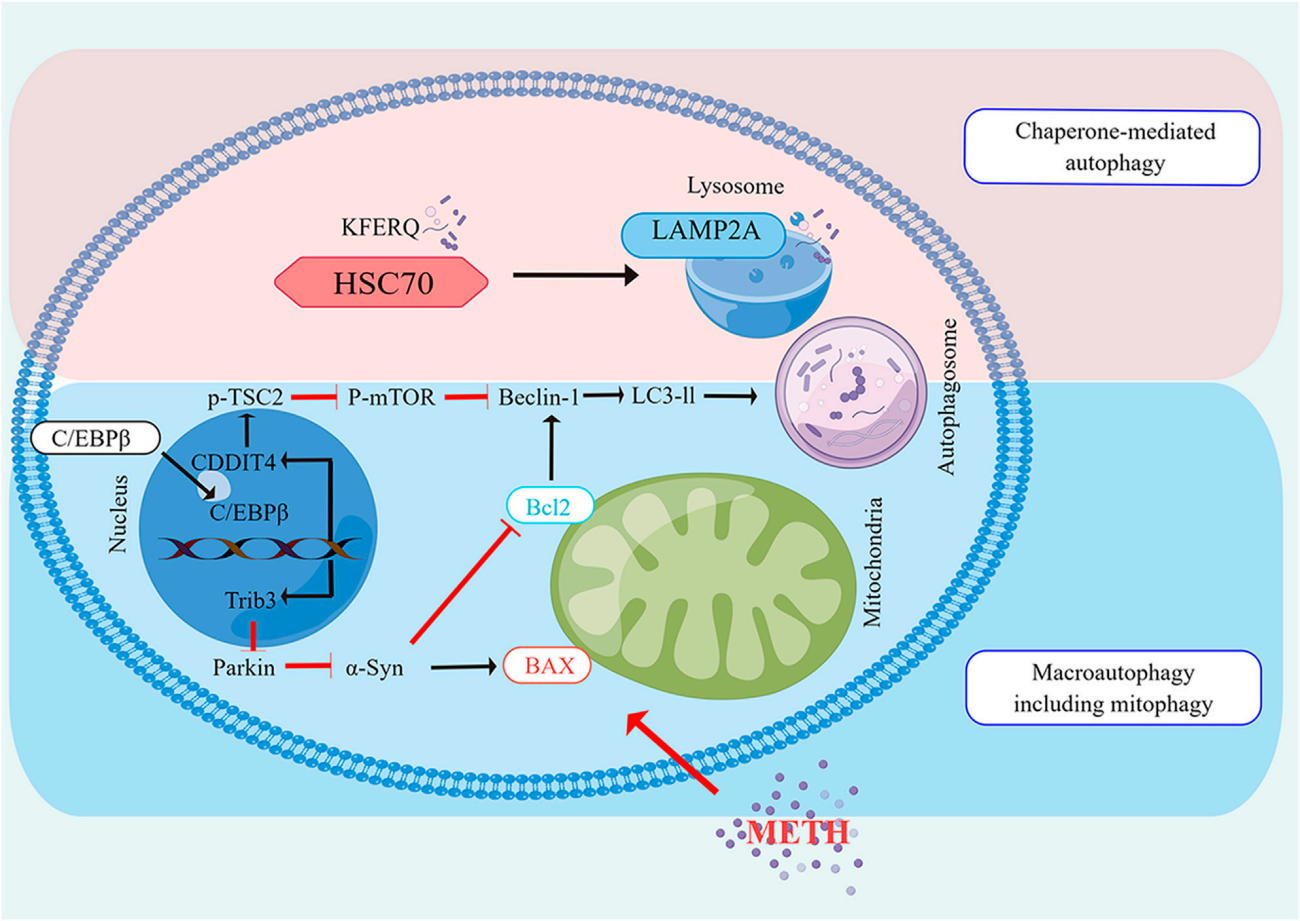
The Molecular pathways of autophagy induced by METH Neurotoxicity The CMA and macro autophagy are both enhanced by METH. Beclin1 is a vital protein in both autophagy pathways that are associated with Bcl2. Macro autophagy is induced by METH via C/EBPβ, either by induction of C/EBPβ/DDIT4 /TSC2/m TOR or by induction of C/EBPβ/Trib3/Parkin/α-Syn. In the CMA induced by METH, the chaperone, HSC70, detects a KFERQ-like motif in the cargo protein. Lipid complexes with the LAMP2A. Translocation of LAMP2A through the lysosomes follows its assembly. Upon entering the lysosome, the substrate protein undergoes rapid degradation by proteases, and then HSC70 is released from the lysosome to bind to another substrate.

Larsen et al. (2002) initially reported METH-induced autophagic changes by demonstrating the formation of autophagic granules when the drug was administered. Specifically, they showed that METH stimulated autophagy granule formation in neuronal varicosities and then in dopaminergic neuron bodies (Larsen et al., 2002). According to their findings, dopamine cannot be effectively sequestered by synaptic vesicles, which promotes autophagy. A later study by Fornai found that METH caused intracellular inclusions in the nuclei of striatal and substantia nigra neurons although no inclusions were observed in frontal cortex neurons (Fornai et al., 2004). It is interesting to note that METH was also able to induce autophagy in a mouse cell line derived from the atrial cardiac chamber. Furthermore, METH treatment resulted in a massive, temporary vacuolization of the cytoplasm followed by an accumulation of granules within the cells (Funakoshi-Hirose et al., 2013). PC12 cells were shown to undergo both autophagy and apoptosis when treated with METH, similar to previous studies (Li et al., 2012). Using taurine reversed its autophagy-inducing effects by restoring mammalian target of rapamycin phosphorylation (p-mTOR) (Li et al., 2012). Additionally, researchers reported that high doses of METH injected into rat striatum resulted in increased expression of autophagy markers such as Beclin-1 and LC3-II (Xu et al., 2018). In a recent study by Subu et al. (2020), stress-induced changes in parameters of autophagy and neuronal apoptosis were observed in the dorsal horn of rats with high levels of METH ingestion. Additionally, Li et al. (2017) reported that METH exposure caused the upregulation of Beclin-1 and LC-II as well as increased DNA damage-inducible transcript 4 (DDIT4) expression. AKT-mTOR signaling can promote human neuroblastoma cell Beclin-1 expression after large doses of METH are administered, as shown by Yang et al. (2019) DDIT4 and AKT are both negative mTOR regulators, which are required for autophagosome formation (Wang et al., 2012; Miao et al., 2021). Intriguingly, C/EBPβ appears to activate DDIT4/TSC2/mTOR or Trib3/Parkin/alpha-synuclein signaling to promote METH-induced autophagy (Krasnova et al., 2013; Xu et al., 2018). Li et al. (2018) have also suggested glycogen synthase kinase3β (GSK3β) produces a marked effect in autophagy and neurodegeneration induced by METH by phosphorylation of Tau and Syn, accumulation of Syn, inhibition of lysosomal degradation, and apoptotic cell death (Figure 2). The impairment of autophagy may also result from METH use (Lin et al., 2012; Funakoshi-Hirose et al., 2013). Therefore, this may be a new mechanism that mediates METH toxicity or protects cells from METH toxicity. A consensus has not yet been reached as to whether autophagy is pro-survival or pro-death during METH-induced toxicity. Larsen et al. (2002) also revealed that pharmacological and genetic intervention inhibiting autophagy from both dopaminergic cell lines decreased their viability during METH exposure, showing that autophagy plays an important role in the survival. In a similar study, Pitaksalee et al. (2015) in SH-SY5Y neuroblastoma cells found that autophagy protected against METH-induced toxicity. When autophagy inhibitors such as 3 MA and wortmanin were administered to cells, cell viability was reduced with an increase in cleaved caspase-3 levels. However, they showed that caffeine reduced METH-induced autophagy. This was demonstrated by a decrease in LC3-II expression and an increase in caspase-3 activation (Pitaksalee et al., 2015). These studies suggest that mTOR or GSK3-βinhibitors, in addition to AMPK/TFEB inducers can reverse DA-mediated behavioral sensitization, memory impairment and morphological alterations (Beaulieu et al., 2004; Castino et al., 2008; Ago et al., 2012; Li et al., 2017; Lazzeri et al., 2018; Yan et al., 2019; Blum et al., 2021). Autophagy appears to modulate DA-related behavior, and therefore is a key therapeutic mechanism for reducing behavioral sensitization caused by METH. A key function of autophagy is to prevent neuro-inflammation and disruption of the blood-brain barrier, closely associated with early effects of DA-related METH administration. Lysosome-associated membrane protein type 2A (LAMP) is a receptor located on the lysosome membrane that plays a crucial role in the CMA (Sun et al., 2019). CMA is limited by LAMP-2A, which forms a complex with heat shock protein 70 kD (hsc70) (Cuervo and Dice, 1996). When human neuroblastoma cell lines (PC12), and primary mouse neurons were exposed to METH, LAMP-2A expression increased as a function of time and dose (Sun et al., 2019). Inhibiting LAMP-2A worsened METH-induced cell death *in vitro* models, suggesting that CMA may act as a protective mechanism in the process. However, it is unknown how CMA could contribute to METH’s neurotoxic effects *in vivo*.

Several previous studies have documented the importance of mTOR in METH-induced autophagy (Kongsuphol et al., 2009; Li et al., 2012). As a result of these studies, METH was found to decrease the level of Phosphorylated-mTOR. It is widely believed that this target plays an important role in cell proliferation and growth (Chen et al., 2016). In addition to p-mTOR, p70 ribosomal S6 protein kinase (p70S6K) and eIF4E binding protein (4EBP1) are also phosphorylated by p-mTOR. A reduction in 4EBP1 phosphorylation induces autophagy, according to research (Corcelle et al., 2009). According to reports, METH also decreased 4EBP1 phosphorylation Chen et al., 2017. DDIT4 is a different target, however, that takes part in autophagy by inhibiting the phosphorylation of mTOR. It has been shown recently that METH promotes autophagy by activating DDIT4 (Ichimura et al., 2000; Chen et al., 2016; Li et al., 2017). Furthermore, decreased mTOR activity enhances autophagosome formation, resulting in autophagy (Ichimura et al., 2000). Autophagosomes are formed by two complexes: the first is composed of Atg6 (Beclin 1), class III PI3K, and Atg14, and the second is composed of Atg12, Atg16, Atg5, and Atg7 (Martini-Stoica et al., 2016). As a result, it has a crucial role to play in recruiting Atg8 (LC3). Exposure to METH activated the Kappa opioid receptor, which elevated Beclin 1 and LC3 II as biomarkers of autophagy (Martini-Stoica et al., 2016). Several studies have demonstrated that Bcl-2 reduces autophagy by interacting with Beclin 1. Therefore, it is assumed that apoptosis and autophagy interact through the creation and dissociation of the Bcl-2/Beclin 1 complex. METH dissociates the Bcl-2/Beclin 1 complex, which is responsible for cells’ survival and death (Nopparat et al., 2010). By phosphorylating and activating JNK1 (c-Jun-N-terminal kinase 1), METH increases autophagy and dissociates Bcl-2/Beclin 1 complexes. Possibly, this mechanism explains why METH seems to have pro-survival rather than death-promoting effects in various studies.

### 2.3 Necroptosis and METH neurotoxicity

Necroptosis is a lytic form of PCD caused by several molecular pathways, which is a loss of plasma membrane integrity, swelling of organelles, and leaking of intracellular contents (Holler et al., 2000). Pharmacological agents or viruses that inhibit caspase-8 can cause necroptosis when TNFR1, TLRs, and other receptors are stimulated (Tummers and Green, 2017). Necroptosis is defined as necrotic cell death that is regulated by receptor-interacting kinase 1 (RIP1), receptor-interacting kinase 3 (RIP3), and the pseudokinase Mixed Lineage Kinase Domain-like (MLKL) (Galluzzi et al., 2014).

**FIGURE 3:**
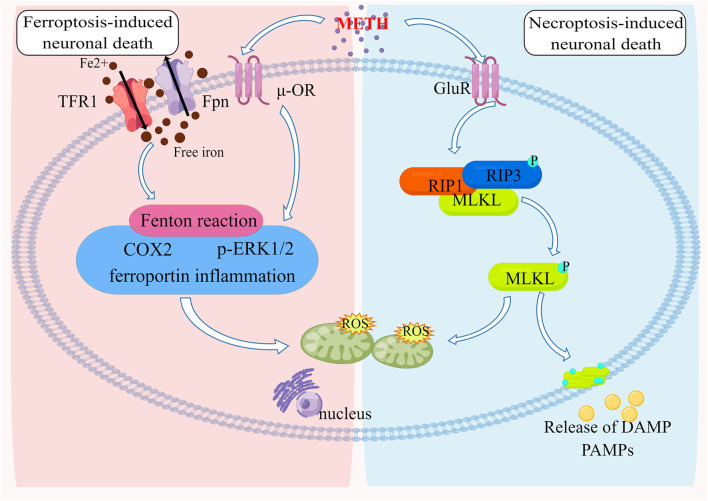
The Molecular pathways of ferroptosis and necroptosis induced by METH Neurotoxicity METH-induced neurotoxicity results from iron overload by modulating the expression of Tfr1 and Fpn. Furthermore, mitochondrial oxidative damage caused by the Fenton reaction is accompanied by ferroportin-induced inflammation as well as ERK1/2 and COX2 upregulation. A reduction or deficiency in GPx4 is associated with mitochondrial energy metabolism that exacerbates injury due to oxidative stress. METH can stimulate the formation of a complex between RIP3 and RIP1, resulting in RIP3 being phosphorylated. Once activated, RIP3 causes MLKL oligomers to form, which cause cell membrane disruption and mitochondrial oxidative damage, resulting in neuronal death.

Xiong et al. (2016) determined that rats’ cortical neurons succumbed to METH-induced necroptosis, the viability of neurons was decreased in a dose and time-dependent manner, and cortical neurons exhibited signs of necroptosis when treated with 1 mM METH at 39°C (Guo et al., 2020). Both METH-poisoned victims had body temperatures above 39 °C before death, accompanied by high expressions of RIP3 and MLKL on neurons (Guo et al., 2020). METH causes hyperthermia (HT), which typically lasts several hours after a single medium or high dose (Tata and Yamamoto, 2007; Krasnova and Cadet, 2009). In addition to aggravating the oxidative and excitotoxic effects of METH, HT leads to an increase in neuron death (He et al., 2004; Chauhan et al., 2014). Based on these findings, it may be that METH and hyperthermia work synergistically to upregulate the expression of RIP3 and MLKL in human cortical neurons. In this study, Guo et al. (2020) examined the effects of methamphetamine at 39°C on embryonic Sprague-Dawley rat cortex neurons and found that methamphetamine at 39°C triggered obvious necrosis-like death in cultured primary cortical neurons, which could be partially inhibited by RIP1 inhibitor Necrostatin-1. With the treatment time of methamphetamine at 39°C extended, the expression of RIP3 and MLKL increased in response to these stimuli. A RIP3 inhibitor GSK872 and propidium iodide staining as well as lactate dehydrogenase release detection demonstrated a significant reduction in neuronal necrosis (He et al., 2004). RIP3 and MLKL protein expression significantly decreased. As a result, METH at 39°C can induce RIP3/MLKL-mediated necroptosis, resulting in neurotoxicity (He et al., 2004). Additionally, Ares-Santos’ experiments demonstrate that METH-treated neurons showed obvious necrotic phenotypes (Ares-Santos et al., 2014). Researchers reported that METH exposure caused neuronal necroptosis in human and mouse striatums largely mediated by the RIP3/MLKL/Drp1 pathway (Zhao et al., 2021) (Figure 3).

### 2.4 Pyroptosis and METH neurotoxicity

Inflammatory pyroptosis is a form of PCD (Cookson and Brennan, 2001). Gasdermins (GSDMs) is composed of six protein subtypes including GSDMA, GSDMB, GSDMC, GSDMD, GSDME, and PJVK(Rogers et al., 2019). These proteins contain a gasdermin-N terminal domain that induces pyroptosis by intrinsic action, which is inhibited by gasdermin-C terminal activity. The gasdermin-N terminal domain is cleaved protease-dependently to enable it to translocate and form oligomers in the plasma membrane, as a result, membrane-spanning pores form, causing a release of cell contents (Ding et al., 2016; Rogers et al., 2019). Both GSDMD and GSDME are cleaved by caspases. GSDMD is the first confirmed protein to be involved in the pyroptotic process. It is cleaved by both caspase-1 and caspase-11 (caspase-4/5) in humans (Kayagaki et al., 2015; Shi et al., 2015). By activating caspase-1, the canonical inflammasome signaling process regulates the maturation process of IL-1β and IL-18 (Raupach et al., 2006). Caspases-11 is activated by non-canonical inflammasome signaling, leading to two distinct cell-intrinsic reactions, pyroptosis induction and caspase-1 activation by NLRP3 (Kayagaki et al., 2015). GSDME is cleaved and activated exclusively by caspase-3 (Wang et al., 2017). Claimed GSDMD-N terminal domain is binding to phosphoinositide in the plasma membrane, causing cellular swelling by forming membrane pores. Eventually, the content leaks and is lysed (Shi et al., 2017). Pyroptosis is often considered a form of apoptosis that is specific to monocytes, as it manifests a blebbing appearance in the plasma membrane. Pytoptosis, however, has been redefined as necrotic cell death due to the discovery of GSDMD and its activity in pore formation. In chronic users of METH, the expression of NLRP1 and NLRP3 has been reported to increase in the hippocampal region indicating pyroptosis (Mahmoudiasl et al., 2019). METH can induce GSDME-induced pyroptosis in the hippocampal of mice, and this process can be regulated by endoplasmic reticulum stress (Liu et al., 2020). (Fan et al., 2022) recently found that the neurotoxicity induced by METH treatment may be achieved through the NLRP1/caspase1/GADMD signaling pathway, and the use of NLRP1 inhibitors can significantly inhibit METH-induced cognitive dysfunction in mice. There is a possibility that drugs that block pyroptosis may reduce the damage caused by METH on hippocampal neurons (Figure 1).

### 2.5 Ferroptosis and METH neurotoxicity

Ferroptosis was first proposed in 2012 by Dr. Brent R. Stockwell of Columbia University (Dixon et al., 2012). It is a new type of programmed cell death in which iron is required (Dixon et al., 2012). Morphologically, iron cells exhibit typical necrotic-like effects, such as swelling and ruptured plasma membranes. Iron cells are characterized by membrane hemorrhaging and shrinkage, as opposed to apoptotic cells. Biochemically, ferroptosis is characterized by lethal levels of iron-dependent lipid peroxidation, leading to complete cellular failure (Xie et al., 2016; Stockwell et al., 2017). Cellular cystine transporters are inhibited (such as erastin), intracellular glutathione (GSH) is depleted, and the inactivation of glutathione peroxidase (GPX4) can cause lipid peroxidation accumulation which could induce cell death to a certain extent (Yang et al., 2014). Similarly, the inhibition of GPX4 enzymes (such as RSL3) can also directly lead to this effect (Yang et al., 2014). Several biological processes are closely involved with ferroptosis, including amino acid, iron, polyunsaturated fatty acid, glutathione, phospholipid, NADPH metabolisms, and coenzyme Q10 biosynthesis (Bersuker et al., 2019; Kraft et al., 2020). Iron chelators can inhibit this process, and many drugs that inhibit or alleviate lipid peroxidation can inhibit the process of ferroptosis, including lipophilic antioxidants, lipid peroxidation inhibitors, phagocytosis inhibitors, etc., The mitochondria, with their iron content and ability to produce ROS, are considered a major site for ferroptosis. Its fatty acid metabolism also provides specific lipid precursors for cellular ferroptosis.

In recent studies, it has been determined that normal iron levels are essential for the development and functioning of the CNS (Pelizzoni et al., 2008; Kellermier et al., 2009; Millward et al., 2013). Ferroptosis is associated with aging, neurodegenerative disease, and failure to respond to drugs or therapies (Ghadery et al., 2015; Li et al., 2017; Viswanathan et al., 2017). According to Li et al. (2017), morphine tolerance is a result of spinal cord ferroptosis. Liroxstatin-1, an inhibitor of ferroptosis, reduces iron overload by increasing the expression of transferrin receptor protein 1/ferroportin and reduces morphine tolerance by increasing GPX4 (Chen et al., 2019). Meanwhile, the reduction of malondialdehyde and reactive oxygen species could be achieved. These findings suggest that morphine tolerance could be treated using ferroptosis. Although the addiction mechanisms of morphine and METH are not identical, inhibition of ferroptosis can still serve as a reference for preventing neuronal damage caused by METH.

Almost all basic cellular processes are dependent on iron homeostasis. It transits between two states of oxidation, ferric iron, and ferrous iron, to perform its diverse functions (Beard et al., 1996). As free iron is lethal to cells, especially at high concentrations, the majority of circulating iron is in the protein-bound ferric form (Stohs and Bagchi, 1995). In particular, free iron is a catalyst for the generation of free radicals through the Fenton reaction (Stohs and Bagchi, 1995; Beard et al., 1996). Therefore, it has a complex set of regulatory mechanisms controlling iron uptake and redistribution processes to safely sequester iron in the cellular matrix while ensuring that the body’s vast demand for iron can be met on a systemic and cellular level (Anderson and Frazer, 2017; Morris et al., 2018). Several neurological processes are influenced by brain iron, including the regulation of dopamine, the production of myelin, and the transport of oxygen in the blood (Daubner et al., 2011). Because of this, iron homeostasis is not only vital for healthy brain development and aging, but also has a major impact on psychostimulant behavior. Iron overload leads to ferroptosis (Morris et al., 2018; Uranga and Salvador, 2018), brain iron accumulation, chronic inflammation (Fox et al., 2012), and increased blood-brain barrier (BBB) permeability (Sajja et al., 2016; Bachi et al., 2017), which are associated with cocaine use disorders, this suggests that ferroptosis exists in cocaine addiction. In fact, this observation is in line with recent studies that show that brain iron levels increase in certain regions of the basal ganglia after extended exposure to psychostimulants, including prescription amphetamine/methylphenidate in attention-deficit/hyperactivity disorder and methamphetamines in vervet monkeys (Melega et al., 2007; Adisetiyo et al., 2014) (Figure 3).

## 3 Potential therapeutic implications for METH neurotoxicity

Most methamphetamine-induced neuropsychiatric consequences can be attributed to its neurotoxicity. Dopaminergic and serotonin receptors are damaged directly and there are neurodegenerative changes, activation of neuronal autophagy, apoptosis, necroptosis, pyroptosis, and ferroptosis (Davidson et al., 2001; Yu et al., 2015; Sanchez et al., 2018; Chen et al., 2019; Liu et al., 2020). In recent years, PCD has made important progress in explaining the complex mechanisms of methamphetamine-induced neurotoxicity. Specifically, the brain’s PCD changes are highly correlated with the symptoms induced by METH, according to *in-vivo* studies. These include cognitive impairment, memory impairment, motor sensitization, and psychosis (Shi et al., 2017). Given the devastating social and public health burden of methamphetamine abuse, further exploration of drug therapy should be highly anticipated.

Luteolin, a flavonoid and an active ingredient in many traditional Chinese medicines, can attenuate neuroinflammation and regulate autophagy in Parkinson’s disease. It can also attenuate Aβ oligomer-induced neuronal responses in Alzheimer’s disease, reduce traumatic brain injury, and exerts neuroprotection against ischemia-induced cellular damage (Xu et al., 2014; Siracusa et al., 2015; Pate et al., 2017; Xu et al., 2017). The researchers discovered that luteolin prevented METH-induced neurotoxicity by inhibiting the PI3K/AKT pathway, decreasing p53 accumulation, and reversing p53-mediated apoptosis and autophagy (Tan et al., 2020). Among the main bioactive components of Gastrodia elata, also known as “Tian ma,” is Gaststrodin, which has remarkable therapeutic value for neurological disorder diseases (Zhan et al., 2016)*.* Gastrodin attenuates Aβ-induced neurotoxicity and microglial activation in Alzheimer’s disease models (Hu et al., 2014)*.* It can inhibit drug-induced apoptosis in numerous experimental and *in vivo* models of Parkinson’s disease and oxidative stress, and protect astrocytes from lipopolysaccharide-induced autophagy (Wang et al., 2014; Wang et al., 2016)*.* According to Yang et al. (Ma et al., 2020), gastrodin supplementation hindered METH-induced expression of LC3-II and Beclin-1, as well as reduced auto phagosome numbers. In addition, gastrodin can also inhibit methamphetamine-induced apoptosis by modulating the c AMP/PKA/CREB pathway in cortical neurons. Curcumin is a natural polyphenol extracted from the L rhizome of turmeric that prevents toxic substances that acting on the nervous system. Curcumin has a powerful autophagy activator effect. With the activation of curcumin, LC3 penetrates autophagy vacuoles, allowing them to enter the cell for clearance and promote autophagy flux, thereby counteracting the neurotoxicity of methamphetamine in the rat pheochromocytoma PC12 cell line (Ryskalin et al., 2021)*.* There is a stilbene type of natural polyphenol known as resveratrol that is capable of entering the brain and exerting powerful neuroprotective effects (Chen et al., 2017; Baghcheghi et al., 2018). Resveratrol protects dopaminergic neurons from methamphetamine-induced neuronal cytotoxicity *in vitro*. In the hippocampus after METH exposure (Kanthasamy et al., 2011; Sun et al., 2015), Zeng et al. (2021) found that pretreatment with resveratrol had a significant effect on inhibiting Bcl-2 expression and increasing Bax and cleaved caspase-3 expression. In addition, resveratrol can also increase cell viability and retard neuronal apoptosis by attenuating METH-induced ROS production and intracellular free calcium overload (Zeng et al., 2021). Inorganic compounds, such as cinnamic aldehyde (CA), are the most effective in fighting neurological diseases, such as Parkinson’s and Alzheimer’s*,* while trans-cinnamaldehyde (TCA) is active in the central nervous system potent anti-inflammatory activity (Chao et al., 2008; George et al., 2013)*.* By inhibiting apoptotic DNA fragmentation, and reducing ROS generation and glutathione levels in PC12 cells, Roughayeh et al. (Rashidi et al., 2021) found that TCA exhibited significant neuroprotective effects against methamphetamine-induced cytotoxicity. The polyphenols found in tea leaves have been shown to have good antioxidant properties, both *in vitro* and *in vivo*. In PC12 cells, tea polyphenols reduced oxidative stress and prevented DNA damage induced by methamphetamine (Ru et al., 2019). Methamphetamine-induced cytotoxicity is markedly neuroprotective-induced neuronal apoptosis, and this process is mediated through the mitochondrial pathway (Ru et al., 2019)*.* Reginsenoside is a major ginsenoside that has been shown to restore nitric oxide (NO) signaling in PTEN-induced putative kinase 1(PINK1) null dopaminergic neuronal cells. Researchers have demonstrated that ginsenoside Re attenuates MA-induced degeneration of dopaminergic neurons *in vivo*, specifically, mitochondrial malfunction, neuroinflammation, and mitochondrial oxidative stress are prevented, as well as PKCδ mitochondrial translocation (Nam et al., 2015)*.* Crocin is a carotenoid compound that has been shown to inhibit superoxide dismutase (SOD) and hydrogen peroxide in oxidative stress neurodegenerative diseases by reducing lipid peroxidation and increasing the activity of antioxidant enzymes enzyme levels (Hosseinzadeh et al., 2005; El-Beshbishy et al., 2012). Shiva Mozaffari et al. found that Crocin ameliorated methamphetamine-induced neuronal apoptosis, oxidative stress, and inflammation in the rat hippocampus via the CREB/BDNF pathway (Mozaffari et al., 2019).

Some clinical drugs for the prevention of neurological diseases have also been used to block methamphetamine-induced neurotoxicity. Antipsychotics such as olanzapine and risperidone can block METH’s impact on the mPFC extracellular glutamate levels and prevent the cell’s apoptosis (Abekawa et al., 2008). Lamotrigine (LTG), an anticonvulsant and mood stabilizer, studies report that it decreases the severity of mitochondrial damage caused by rotenone or MPP+, in turn, caspase-3 is activated, oxidative stress is increased, and cell death is induced (Kim et al., 2007). Despite repeated administration of LTG and METH, METH was unable to induce TUNEL-positive cells in mPFC (Lizasoain et al., 1995). In addition, it prevents NO production from being enhanced (Bashkatova et al., 2003). Thus, not only does LTG block the METH-induced delayed increase in mPFC extracellular glutamate levels, but also LTG blocks the development of apoptosis in this region. Among the most effective treatments for mild to moderate Alzheimer’s disease is memantine (MEM), a non-competitive antagonist of the NMDA receptor (Waite, 2015). MEM pretreatment was able to reverse METH-induced changes in Bcl-2 and caspase-3 apoptosis-related gene protein levels in PFC, in addition, MEM induced no cognitive deficits in METH-induced mice, which suggests MEM’s effectiveness may be due to its anti-apoptotic activity (Long et al., 2017).

Interventions for ferroptosis, iron chelator deferiprone has been used in targeting iron therapeutic strategies recently (Crapper McLachlan et al., 1991). According to studies, deferiprone inhibits ferroptosis *in vitro* and appears to be effective against Alzheimer’s disease, Parkinson’s disease, and Amyotrophic Lateral Sclerosis in mice (Guo et al., 2013; Zhang and He, 2017). By using the Unified Parkinson’s Disease Rating Scale (UPDRS) to measure the progression of symptoms, deferiprone significantly impacted brain iron levels and slowed or delayed the progression of symptoms in Parkinson’s disease (Zhang and He, 2017). Using a cell-free lipid peroxidation system or RSL3 or erastin *in vitro*, Cu^II^ (atsm) strongly inhibits ferroptosis induced by lipid peroxidation (Nikseresht et al., 2020). The efficacy of Cu^II^ has been extensively tested (and independently validated) in preclinical animal models, including models of Amyotrophic Lateral Sclerosis, Parkinson’s disease, and stroke (Nikseresht et al., 2020). Results of clinical trials on Amyotrophic Lateral Sclerosis and Parkinson’s disease have been encouraging. Neuronal damage caused by methamphetamine and pathogenic causes of Alzheimer’s disease and Parkinson’s disease is caused by abnormal dopamine metabolism (Nikseresht et al., 2020). Therefore, METH-induced neurotoxicity may also be impacted by these ferroptosis interventions.

Although abundant research has been conducted to explore the prevention and treatment of methamphetamine-induced neuronal death, it still requires some effective novel drug therapies for preclinical and clinical studies. Multiple pathways of programmed cell death are activated by methamphetamine abuse. Current research focuses on blocking a single pathway with a single drug. Methamphetamine-induced neurotoxicity can be complicated, making multi-compound drugs that target multiple pathways more effective.

## 4 Conclusion and perspectives

Defects in processes of PCD have been implicated in METH-induced neurotoxicity. However, it has not been clearly defined whether these defects are the real cause or a key factor of METH addiction. Although blocking the cell death effectively, it still fails to provide an effective and qualified treatment. In conclusion, the authors describe the pathogenic mechanisms of PCD on METH-induced neurotoxicity, such as DA release, oxidative stress, mitochondrial dysfunction, and caspase cascade. We still need a better understanding of cell death in METH-induced neurotoxicity in order to achieve revolutionary progress.

## Data Availability

The original contributions presented in the study are included in the article/Supplementary Material, further inquiries can be directed to the corresponding author.
